# Fas-associated factor 1 inhibits tumor growth by suppressing Helicobacter pylori-induced activation of NF-κB signaling in human gastric carcinoma

**DOI:** 10.18632/oncotarget.14033

**Published:** 2016-12-20

**Authors:** Ai-qun Liu, Zhongqiu Xie, Xiao-ni Chen, Jie Feng, Jia-wei Chen, Fu-jun Qin, Lian-ying Ge

**Affiliations:** ^1^ Department of Endoscopy, The Affiliated Tumor Hospital of Guangxi Medical University, Nanning 530021, Guangxi, P.R. China; ^2^ Department of Pathology, School of Medicine, University of Virginia, Charlottesville, VA 22908, USA

**Keywords:** gastric cancer, Fas-associated factor 1, NF-κB, Helicobacter pylori

## Abstract

Loss of Fas-associated factor 1 (FAF1) may act as a pro-survival signal in diseased cells, but whether this is true in gastric carcinoma remains unclear. Here we report that FAF1 was expressed at low levels in gastric carcinoma tissues and cell lines, and its expression correlated with larger tumors, higher histology grade, higher TNM stage, tumor infiltration, and lymph node metastasis. Univariate analysis and survival curve analysis identified low FAF1 expression as a predictor of poor prognosis. FAF1 overexpression in HGC-27 gastric cancer cells induced cell apoptosis and inhibited cell proliferation and growth. It also reduced colony formation *in vitro* and tumor growth in mice. We found that *Helicobacter pylori*, a risk factor for gastric cancer, down-regulated FAF1 expression via NF-κB signaling. Knock-down of IKKβ or p65 expression in gastric cancer cells reversed *H. pylori*-induced down-regulation of FAF1 expression and partially blocked *H. pylori*-induced secretion of inflammatory cytokines TNF-α and IL-8. Our results suggest that loss of FAF1 contributes to human gastric carcinogenesis by allowing H. pylori to activate NF-κB signaling.

## INTRODUCTION

Gastric cancer is the fourth most common human cancer in the world, with approximately 934,000 new cases and 73,800 deaths occuring every year. More than 70% of gastric cancer cases occur in developing countries, with nearly half of all cases occurring in China [1]. Despite rapid advancements in surgery, chemotherapy, and radiation therapies for gastric cancer, 5-year overall survival remains around 29% because of tumor recurrence and distant metastases [2]. Therefore, there is an urgent need to reveal the molecular mechanisms underlying gastric carcinoma in order to develop novel therapeutic strategies.

Fas-associated factor 1 (FAF1), a 74-kDa pro-apoptotic protein also known as Fas antigen, is involved in diverse biological processes [3], and its loss has been reported in uterine cervical carcinoma [4], mantle cell lymphoma [5] and malignant mesothelioma [6]. In previous work, we showed that FAF1 mRNA levels are lower in gastric cancer tissue, especially in poorly differentiated tumors, than in healthy gastric tissue [7]. These results suggested that FAF1 plays a role in the progression of gastric carcinogenesis.

Further evidence for this idea comes from the association between FAF1 expression and infection with *Helicobacter pylori*, a Group I carcinogen in gastric carcinogenesis [8]. *H. pylori* infection is strongly linked to the development of gastritis, peptic ulcers, gastric atrophy and gastric cancer [9]. Our laboratory has shown that among gastric cancer patients, FAF1 mRNA levels are lower in tissues positive for *H. pylori* than in tissues negative for *H. pylori* [7]. *H. pylori* may increase risk of gastric cancer in part by activating NF-κB signaling [10, 11], leading to secretion of pro-inflammatory cytokines such as tumor necrosis factor-α (TNF-α), interleukin-1 (IL-1β), interleukin-6 (IL-6) and interleukin-8 (IL-8). Therefore we wondered whether FAF1 may be affected by NF-κB signaling.

To gain deeper insights into the potential role of FAF1 in gastric cancer, we determined levels of FAF1 expression in gastric carcinoma tissues and cell lines, and we examined the effects of FAF1 overexpression on tumor cell proliferation *in vitro* and tumor growth *in vivo*. We retrospectively analyzed clinical data in order to identify possible relationships of FAF1 expression with patient or disease characteristics or with prognosis. Finally, we examined whether FAF1 may influence gastric carcinogenesis by inhibiting *H. pylori*-induced NF-κB activation.

## RESULTS

### Low FAF1 expression characterizes human gastric carcinoma and predicts poor prognosis

**Figure 1:**
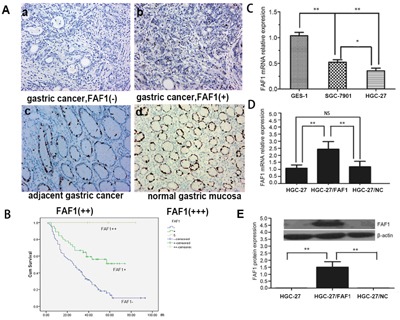
FAF1 is expressed at low levels in gastric cancer tissue, and it predicts poor prognosis **A**. Expression of FAF1 protein in gastric cancer patients with immunohistochemistry, showing (a) negative expression in cancer tissue, (b) weakly positive expression in cancer tissue, (c) protein expression in adjacent tissue and (d) intense positive expression in normal tissue. Magnification, ×200. **B**. Overall survival rate was significantly lower in patients with low or negative FAF1 expression after radical resection than in those with high FAF1 expression, based on the log-rank test. **C**. Relative levels of FAF1 mRNA measured by RT-PCR in GES-1, SGC-7901 and HGC-27 cell lines. Levels were significantly lower in HGC-27 and SGC-7901 cells than in GES-1 cells. **D**. Quantitative RT-PCR showed higher FAF1 mRNA levels in HGC-27/FAF1 cells than in HGC-27/NC or HGC-27 cells. **E**. Western blotting showed higher FAF1 protein levels in HGC-27/FAF1 cells than in HGC-27/NC or HGC-27 cells. Data are mean ± SD of three independent experiments (n = 3). *P < 0.05, **P < 0.01. NS, P > 0.05.

FAF1 protein expression was measured using immunohistochemistry in samples of gastric cancer tissue, adjacent tissue and normal gastric tissue from 145 patients with gastric cancer. FAF1 was weakly expressed in gastric cancer tissue, but strongly expressed in adjacent tissue or normal gastric tissue (Figure 1A a–d). Comparison of FAF1 expression with clinicopathological characteristics (Table 1) identified negative associations of FAF1 expression with tumor size (P = 0.024), histology grade (P < 0.01), tumor infiltration (P < 0.01), lymph node metastasis (P < 0.01), and TNM stage (P < 0.01). Univariate analysis identified the following predictors of survival: FAF1 expression, tumor diameter, tumor location, tumor infiltration, histology grade, lymph node metastasis, distant metastasis and TNM stage (Table 2). Stratification of patients by FAF1 expression and comparison of their survival curves showed lower survival rate among those with low FAF1 expression (Figure 1B).

**Table 1:** Correlation of FAF1 expression with clinicopathologic characteristics in patients with gastric cancer

Parameter	N	FAF1-positive		P
		n	%	
**Gender**				0.751
Male	97	37	38.1	
Female	48	17	35.4	
**Age (years)**				0.494
≦60	86	34	37.3	
>60	59	20	37.0	
**Tumor size (cm)**				0.024
<5	80	36	45.0	
≧5	65	18	27.6	
**Tumor site**				0.642
Cardia	29	10	34.4	
Body	22	8	36.3	
Antrum	76	33	43.4	
Multiple sites	18	3	16.6	
**Histology grade of differentiation**				<0.01
Well-moderate	28	21	75.0	
Poor-undifferentiated	117	33	28.2	
**Tumor infiltration**				<0.01
T1	9	7	77.7	
T2	25	16	64.0	
T3	67	22	32.8	
T4	44	9	20.4	
**Lymph node metastasis**				<0.01
N0	59	32	54.2	
N1	86	22	25.5	
**Distant metastasis**				0.374
M0	130	50	38.4	
M1	15	4	26.6	
**TNM stage**				<0.01
I	27	20	74.0	
II	56	22	39.2	
III	47	8	17.0	
IV	15	4	26.6	

**Table 2:** Univariate Cox regression analysis to identify predictors of survival in patients with gastric cancer after radical surgery

Factor	HR	95% CI	P
Tumor diameter	2.967	1.959-4.494	0.000
Anatomic site	1.343	1.056-1.708	0.016
Tumor infiltration	4.205	2.950-5.995	0.000
Histology grade	3.470	1.735-6.940	0.000
Lymph node metastasis	3.707	2.306-5.960	0.000
Distant metastasis	8.891	4.753-16.632	0.000
TNM stage	5.293	3.856-7.266	0.000
FAF1 expression	0.330	0.209-0.522	0.000

### FAF1 overexpression inhibits gastric cancer cell proliferation and induces apoptosis *in vitro*

Using real-time PCR, we measured FAF1 mRNA levels in the HGC-27 and SGC7901 gastric cancer cell lines as well as in the GES-1 normal gastric epithelial cell line. Levels were much lower in the two cancer lines than in the normal line, and levels were lowest in HGC-27 cells (Figure 1C), so this line was chosen for subsequent experiments.

We engineered a recombinant lentivirus encoding FAF1, with which we infected HGC-27 cells. In parallel, we infected another set of cultures with a negative-control lentivirus encoding no transgene. Using real-time PCR, we found that cells infected with our recombinant lentivirus (HGC-27/FAF1) significantly overexpressed FAF1 relative to uninfected cells (HGC-27) or cells infected with negative-control virus (HGC-27/NC; P < 0.01, Figure 1D). Lentivirus infection on its own did not affect FAF1 expression levels, since levels were similar between HGC-27/NC and HGC-27 cells (P = 0.58, Figure 1D). Consistent with these mRNA results, Western blotting revealed significantly higher FAF1 protein levels in HGC-27/FAF1 cells than in the other two groups (P < 0.01, Figure 1E).

**Figure 2:**
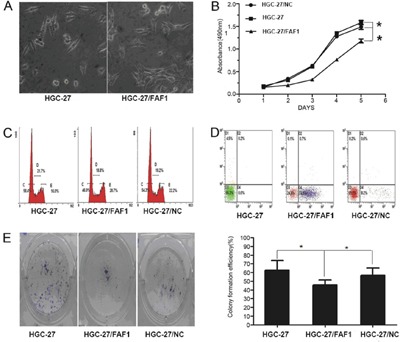
FAF1 overexpression inhibits gastric cancer cell proliferation and induces apoptosis in vitro **A**. Morphological changes in HGC-27 cells as a result of FAF1 overexpression. **B**. Comparison of *in vitro* proliferation of HGC-27, HGC-27/FAF1 and HGC-27/NC cells. **C**. Comparison of cell cycle distribution of HGC-27, HGC-27/FAF1 and HGC-27/NC cultures, based on flow cytometry. **D**. Comparison of apoptosis levels in HGC-27, HGC-27/FAF1 and HGC-27/NC cells, based on flow cytometry. **E**. Comparison of colony formation by HGC-27, HGC-27/FAF1 and HGC-27/NC cells. The left panel shows representative results for the three types of cells. Quantitation of colony numbers is shown in the right panel. Data are mean ± SD of three independent experiments (n = 3). *P < 0.05, **P < 0.01.

We then examined the effects of FAF1 overexpression on key characteristics of gastric cancer cells. Overexpression significantly decreased proliferation (P < 0.01, Figure 2A–2B), increased the proportion of cells in G2/M stage (P < 0.05, Figure 2C), increased the proportion of apoptotic cells (P < 0.05, Figure 2D) and decreased colony formation (P < 0.05, Figure 2E).

### FAF1 overexpression inhibits tumor growth *in vivo*

**Figure 3:**
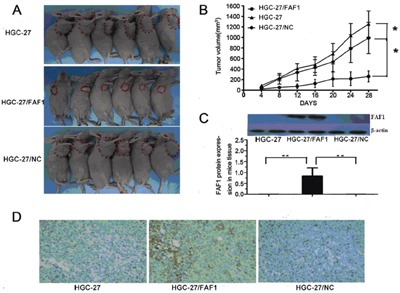
FAF1 overexpression inhibits tumor growth in vivo **A**. Tumor volume was significantly smaller in nude mice injected with HGC-27/FAF1 cells than in mice injected with HGC-27 or HGC-27/NC cells. **B**. Growth of HGC-27/FAF1 tumors in nude mice was significantly slower than growth of HGC-27 or HGC-27/NC tumors. **C**. FAF1 expression in tumor xenografts, based on Western blotting. **D**. FAF1 expression in tumor xenografts, based on immunohistochemistry. Six animals were analyzed for each treatment condition. *P < 0.05, **P < 0.01.

To extend these *in vitro* experiments suggesting that FAF1 suppresses gastric cancer progression, we compared the growth of HGC-27/FAF1, HGC-27/NC or HGC-27 tumors in nude mice. Tumor volume at 28 days after injection was significantly smaller in mice injected with HGC-27/FAF1 cells than in mice injected with the other types of cells (P < 0.05, Figure 3A–3B).

We next used Western blotting to compare levels of FAF1 protein among the three types of tumors. FAF1 expression was high in mice injected with HGC-27/FAF1 cells, but undetectable in mice injected with the other types of cells (Figure 3C). Immunohistochemistry of HGC-27/FAF1 tumor sections showed FAF1 to localize in the cytoplasm (Figure 3D). Little or no immunohistochemical staining was observed for FAF1 in sections of HGC-27/NC or HGC-27 tumors.

### Proteomics-based prediction of effects of FAF1 overexpression on *H. pylori*-associated gastric carcinogenesis

**Figure 4:**
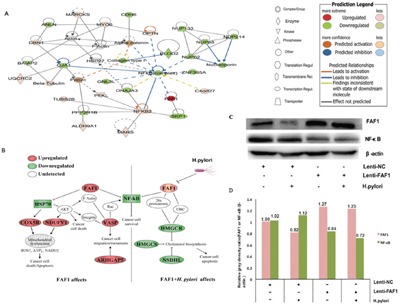
Map of pathways potentially involved in FAF1/H. pylori-associated gastric carcinogenesis based on iTRAQ quantification and Western blotting **A**. Networks of protein inter-relationships were constructed using Ingenuity Pathway Analysis. Red proteins were predicted to be up-regulated, and green proteins to be down-regulated, in FAF1/*H. pylori*-associated gastric carcinogenesis. **B**. Protein interaction networks involving FAF1 and *H. pylori*. Analyzing these networks using the “molecule activity predictor” algorithm in Ingenuity Pathway Analysis generated predictions of the effects of FAF1 up- or down-regulation on downstream molecules. **C**. Western blotting validation of proteomics results based on different combinations of FAF1 overexpression, NF-κB expression and/or *H. pylori* infection. **D**. Image J quantitation of relative FAF1 and NF-κB protein expression in different cell groups. Relative gray density ratios are shown above the bars.

We established a proteomics database of HGC-27/FAF1 cells infected with *H. pylori*, and “molecule activity predictor” analysis predicted that FAF1 up-regulation would inhibit the NF-κB complex at the center of the network. This was corroborated by iTRAQ measurements (Figure 4A). These results suggested that FAF1 overexpression should lead to a net increase in apoptosis of gastric cancer cells, which is consistent with the *in vitro* and *in vivo* experiments described above. Comparison of HGC-27/FAF1 infected or not infected with *H. pylori* showed that infection slightly reduced expression of FAF1 but increased expression of NF-κB, based on iTRAQ and Western blotting of total cell lysates (Figure 4B–4D).

### *H. pylori* may act via IKKβand p65 to down-regulate FAF1

**Figure 5:**
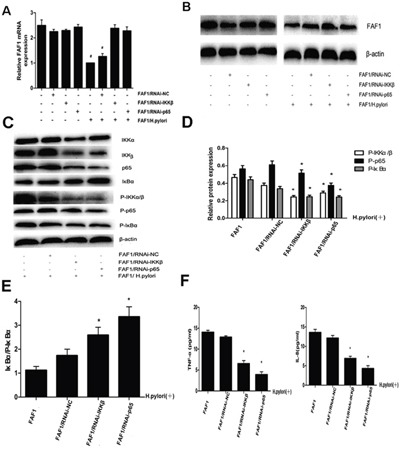
H. pylori down-regulates FAF1 expression via the NF-κB signaling pathway **A**. Quantitative RT-PCR analysis of FAF1 mRNA levels in HGC-27/FAF1 cells not transfected or transfected with negative-control RNAi (RNAi-NC) or with RNAi targeting IKKβor p65. Cells were infected or not with *H. pylori*. *P < 0.05 *vs* untransfected cells or cells transfected with RNAi-NC. **B**. Western blotting of total lysates from HGC-27/FAF1 cells not transfected or transfected with negative-control RNAi (RNAi-NC) or with RNAi targeting IKKβ or p65. Cells were infected or not with *H. pylori*. **C**. Western blotting of proteins in the NF-κB signaling pathway. **D**. Western blotting showed that levels of phosphorylated IKKα/β, p65 and IκBα were lower in RNAi targeting IKKβ or p65 than in untransfected cells or cells transfected with negative-control RNAi (P < 0.05). **E**. The ratio of IκBα to P-IκBα was higher in cells transfected with RNAi against IKKβ or p65 than in untransfected cells or cells transfected with negative-control RNAi (*P < 0.05). **F**. Secretion of TNF-α and IL-8 was lower in *H. pylori*-infected cells transfected with RNAi against IKKβ or p65 than in *H. pylori*-infected cells that were untransfected or transfected with negative-control RNAi (*P < 0.05). Data are the mean ± SD of three independent experiments.

To identify how *H. pylori* infection may down-regulate FAF1 expression, we examined the effects of silencing IKKβ or p65 genes on FAF1 expression in the presence or absence of *H. pylori* infection. These two proteins are key downstream effectors of the NF-κB signaling pathway [12, 13]. HGC-27/FAF1 cells were transfected for 72 h with RNAi constructs targeting IKKβor p65, or with a non-coding negative-control RNAi construct. Then real-time PCR was used to compare levels of FAF1 mRNA in the different groups, while Western blotting was used to compare levels of FAF1 protein. When either IKKβ or p65 was knocked-down, *H. pylori* infection did not significantly down-regulate FAF1 expression at the level of mRNA (Figure 5A) or protein (Figure 5B).

Western blotting further showed that knocking down IKKβ or p65 partially blocked *H. pylori*-induced IκBα degradation and IKKαup-regulation, and substantially blocked *H. pylori*-induced phosphorylation of IKKα/β, p65 and IκBα (P < 0.05, Figure 5C–5D). As a result, these knock-downs increased the level of unphosphorylated IκBαrelative to the level of phosphorylated IκBα (P < 0.05, Figure 5E). Knocking down either IKKβ or p65 also partially blocked *H. pylori*-induced secretion of the inflammatory cytokines TNF-α and IL-8 (P < 0.05, Figure 5F).

## DISCUSSION

Here we investigated the potential involvement of FAF1 in gastric cancer and asked whether NF-κB signaling pathways may mediate this involvement. Analysis of gastric cancer tissues suggests that FAF1 expression is quite low in these patients, and that low FAF1 expression may predict patient survival (Figure 1A–1B and Tables 1–2). Consistent with a protective role of FAF1 against gastric cancer, we found that FAF1 overexpression in gastric cancer cell lines reduced their proliferation and increased apoptosis (Figure 2A–2E). FAF1 overexpression also reduced tumor growth *in vivo* (Figure 3A–3D). Based on the predictions of proteomic reference pathways and networks in the presence of high FAF1 expression and *H. pylori* infection, we identified the NF-κB pathway as a potential mediator of the link between FAF1 and gastric cancer (Figure 4A–4D). Consistent with this idea, knocking down expression of the NF-κB activator proteins IKKβ or p65 blocked the ability of *H. pylori* to down-regulate FAF1 expression as well as trigger other proinflammatory changes linked to gastric cancer (Figure 5A–5F). Taken together, our results suggest that FAF1 normally suppresses gastric carcinogenesis by promoting cell death and apoptosis, as well as by blocking the ability of H. pylori to stimulate NF-κB signaling.

H. pylori virulence factors activate NF-κB [14] via canonical pathways involving activation of an IKK complex containing IKKα and IKKβ [15, 16], phosphorylation and subsequent degradation of IκB, and translocation of p65 to the nucleus, leading to DNA binding by NF-κB [17–19]. Our results suggest that loss of FAF1 expression is related to greater NF-κB activation during gastric carcinogenesis, which is consistent with previous work [20]. Our finding that FAF1 is suppressed by *H. pylori*-activated NF-κB signaling in gastric carcinogenesis, coupled with previous reports that FAF1 inhibits NF-κB activity by interfering with nuclear translocation of p65 [21] and IKKβ [22], suggests a new set of interactions that can be analyzed more deeply to gain more insights into how gastric cancer progresses.

For example, we found here that knocking down key components of NF-κB signaling down-regulated expression of TNF-α and IL-8. This leads us to propose that the up-regulation of TNF-α and IL-8 following *H. pylori* infection of gastric cells may contribute to FAF1 down-regulation.

It is also possible that FAF1 affects gastric cancer progression through mechanisms that do not depend on NF-κB. For example, Bessède et al showed that alterations in IQGAP1 signaling in the presence of H. pylori infection can promote the emergence of cancer stem cells and gastric adenocarcinoma development [23]. H. pylori–induced inflammation leads to high gastric endothelial cell turnover and increased opportunities for DNA damage and somatic mutations [24, 25]. It would be interesting to examine whether FAF1 is involved in any of these processes. FAF1 has been found to promote hydrogen peroxide-mediated necrotic cell death [26]. Whether this contributes to the observed tumor suppressive effects of FAF1 should be investigated.

In conclusion, we have provided evidence that FAF1 acts a tumor suppressor by regulating cell proliferation and apoptosis, and by inhibiting NF-κB signaling--such as after H. pylori infection--that would otherwise stimulate secretion of pro-inflammatory cytokines and increase the risk of gastric carcinogenesis. Our work justifies studies into therapeutic approaches that target pathways that down-regulate FAF1, especially the NF-kB pathway as a result of H. pylori infection.

## MATERIALS AND METHODS

### Tissue samples

A total of 145 gastric cancer samples were obtained from the same number of patients who underwent surgery at the Affiliated Tumor Hospital of Guangxi Medical University between April 2004 and December 2007. Patients were diagnosed with gastric cancer based on histopathology evaluation. None received local or systemic treatment before surgery. All gastric tissue samples were immediately frozen in liquid nitrogen, and stored at −80°C until use. The study protocol was approved by the Research Ethics Committee of the Affiliated Tumor Hospital, and informed consent was obtained from all patients.

### Cell lines and animals

Cell lines were obtained from the National Institute of Cells (Shanghai, China) and the Central Laboratory of Zhongnan University, Hunan, China. RPMI-1640 medium (Gibco, USA) was supplemented with 10% fetal bovine serum (Corning, USA), penicillin (100 U/mL) and streptomycin (0.1 mg/mL). Cells were cultured at 37°C in a humidified atmosphere of 5% CO_2_.

Male nude mice aged 4-6 weeks were obtained from the animal center of Guangxi Medical University (Nanning, China) and fed a standard rodent diet under specific pathogen-free conditions. Animal care and all experimental procedures were approved by the Animal Ethics Committee of Guangxi Medical University.

### Preparation of *H. pylori* culture filtrate

*Helicobacter pylori* strain NCTC11637 was purchased from American Type Culture Collection (ATCC). *H. pylori* was taken from liquid nitrogen, inoculated onto Columbia blood agar plate medium containing 10% defibrinated sheep blood, and incubated at 37°C in a microaerobic environment (5% O_2_, 10% CO_2_, 85% N_2_). After 3 d of culturing, *H. pylori* was transferred to *Brucella* broth medium and incubated with shaking (130 rev/min) for 3 d at under 37°C under microaerobic conditions. *H. pylori* supernatant was prepared by centrifuging cultures at 12000 rev/min for 20 min, concentrating and filtering the supernatant through a 0.22-μm pore size, and storing at −80°C. Vacuolar cytotoxic activity was tested as described [27], with some modifications.

### Construction of lentivirus FAF1 overexpression vectors and RNAi vectors

Lentivirus expression vectors encoding full-length human FAF1 (Lenti-FAF1) and empty control vector (Lenti-NC) were synthesized by Shanghai GeneChem (Shanghai, China). Constructs for RNA interference (RNAi) targeting the human NF-κB p65 subunit mRNA (GenBank, NM_021975) or human NF-κB IKKβ subunit mRNA (GenBank, NM_001190720) were also synthesized by Shanghai GeneChem. As a negative RNAi control, a random non-coding RNA was synthesized (5′-TTC TCC GAA CGT GTC ACGT-3′).

### Cell transfection

HGC-27 cells were cultured in 6-well plates for 24 h, then transfected with Lenti-FAF1 or Lenti-NC overexpression constructs using Lipofectamine® 2000 Reagent (Invitrogen, Thermo Fisher Scientific) according to the manufacturer's instructions. Transfection efficiency was assessed at different time points using fluorescence microscopy. Puromycin (1 mg/mL) was used to select stable transfectants. The resulting stably transfected cell lines were designated HGC-27/FAF1 and HGC-27/NC.

HGC-27/FAF1 cells were cultured in 6-well plates for 24 h, then transfected with RNAi constructs targeting IKKβ or p65 or with the negative-control construct using Lipofectamine® 2000 according to the manufacturer's instructions. At different time points, transfection efficiency was assessed using fluorescence microscopy.

### Prediction of proteins involved in FAF1-regulated interaction networks

To identify molecular details about how FAF1 and *H. pylori* may contribute to gastric carcinogenesis, we used iTRAQ labeling and LC-MS/MS to perform proteomic analysis of HGC-27/FAF1 and HGC-27/NC cells in the presence or absence of *H. pylori* infection. Protein samples were prepared for proteomic analysis as described [28], and iTRAQ 8-plex labeling and analysis were carried out following the manufacturer's instructions (Applied Biosystems, Foster City, CA, USA). The “molecule activity predictor” algorithm of Ingenuity pathway analysis (IPA) was used to predict whether a molecule of interest, pathway, network or function was up- or down-regulated under conditions of FAF1 overexpression and/or *H. pylori* infection. These predictions were then used to infer up- or down-regulation of upstream and downstream proteins.

### Protein extraction and western blotting

Cells or mouse tissues were washed with pre-cooled phosphate-buffered saline (PBS) and lysed in RIPA lysis buffer containing 1 mmol/L phenylmethylsulfonyl fluoride (PMSF) for 30 min on ice. Clarified protein extracts were obtained by centrifugation for 30 min at 4°C at 12000 rev/min. Protein concentration was determined using a BCA protein assay kit (Boster, Wuhan, China). Lysate (100 μg) was mixed with loading buffer containing 5% β-mercaptoethanol, and heated for 5 min at 100°C. Samples were separated by sodium dodecyl sulfate-polyacrylamide gel electrophoresis (SDS-PAGE), and proteins were transferred onto PVDF membrane filters (Millipore, USA) by wet blotting. Membranes were incubated for 1 h at room temperature in blocking buffer (1 × TBS with 0.1% Tween-20 and 5% skim milk), then incubated overnight at 4°C with a primary antibody against FAF1 (1:1500; GeneTex, USA) or a primary antibody against one of the following (Cell Signaling Technology, USA): NF-κB (1:1000), p65 (1:1000), IKKα/β (1:1000), IκBα(1:1000), P-p65 (1:1000), P-IKKα/β(1:1000), P-IκBα(1:1000) andβ-actin (1:1000). Membranes were incubated with the corresponding horseradish peroxidase-conjugated secondary antibody (1:2000; Cell Signaling Technology) for 1 h at room temperature. Finally, membranes were analyzed using an ECL system (Beyotime, Shanghai, China) and quantitated using Bio-Rad Quantity One software 4.62.

### Total RNA extraction and quantitative real-time PCR

Total RNA was extracted from cells or mouse tissue using Trizol (Invitrogen, CA, USA) according to the manufacturer's instructions, and RNA concentration was measured using the Nano Drop 2000 (Thermo Fisher Scientific). RNA template (3 μg) was reverse-transcribed using the first-strand cDNA synthesis kit (Thermo Fisher Scientific). Quantitative real-time PCR was performed in two steps using the MxPro 3000 system and SYBR Premix Ex Taq PCR (Takara, Dalian, China). PCR conditions were as follows: 95°C for 30 sec, followed by 40 cycles of 95°C for 5 sec and 60°C for 34 sec. PCR primers were as follows: FAF1, 5′-CTTGCTGAATCAGGGCTCTC-3′ (forward), 5′-TCCACCCCAAATTCTGTAGC-3′ (reverse); IKKβ, 5′-GGCAAACCGTACTCCAAGCAC-3′ (forward), 5′-CCTTGTCTGCACACTGGAGGTC-3′ (reverse); p65, 5′-GGGAAGGAACGCTGTCAGAG-3′ (forward), 5′-TAG CCTCAGGGTACTCCATCA-3′ (reverse); β-actin, 5′-AC CGAGCGCGGCTACAGC-3′ (forward), 5′-CTC ATTGCCAATGGTGAT-3′ (reverse). Levels of mRNA were calculated based on threshold cycle (Ct) values and normalized to levels of β-actin mRNA. Results were analyzed using the 2^-ΔΔCt^ method and the formula ^ΔΔ^Ct = Sample (Avg · FAF1/IKKβ/p65 Ct - Avg · β-actin Ct) - Control (Avg · FAF1/IKKβ/p65 Ct - Avg · β-actin Ct).

### Cell proliferation assay

Cells (0.5 × 10^4^ per well) were seeded into 96-well plates in a final volume of 100 μl. Every 24 h, 10 μl of Cell Counting Kit-8 solution (CCK-8, Dojindo) was added to each well of one plate, and the plate was further incubated for 2 h at 37°C. Absorbance at 450 nm was measured using a microplate reader (MK3, Thermo Electron Corporation, MA, USA). The experiment was performed over five consecutive days.

### Cell cycle analysis

Cells (1 × 10^6^ per well) were seeded into 6-well culture plates, and transfected with Lenti-FAF1 or Lenti-NC. Cells were allowed to grow to 90% confluence, then treated with trypsin, centrifuged, washed twice with ice-cold PBS, and fixed in 70% cold ethanol at 4°C overnight. Cells were incubated in 100μl RNase A (eBioscience, CA, USA) at 37°C for 30 min, then in 400μl propidium iodide (eBioscience) at 4°C for 30 min. Stained cells were analyzed by flow cytometry (EpicsXL, Beckman, CA, USA) using EXPO32 ADC analysis software.

### Cell apoptosis analysis

Cells (1 × 10^6^ per well) were seeded into 6-well culture plates and transfected with Lenti-FAF1 or Lenti-NC as described above. Cells were allowed to grow to 90% confluence, washed once with PBS, washed once with 1× binding buffer (eBioscience), collected, and resuspended in 1× binding buffer. Fluorochrome-conjugated Annexin V PE (5 μl; eBioscience) was added to 100 μl of cell suspension, and the mixture was incubated for 15 min at room temperature away from light. Then cells were washed with 1× binding buffer, centrifuged, resuspended in 200 μl 1× binding buffer and mixed with 5μl 7-AAD Viability Staining Solution (eBioscience). Finally the suspension was analyzed using flow cytometry as described above.

### Colony formation assay

HGC-27/FAF1 or HGC-27/NC cell lines were cultured for 14 d, then stained with gentian violet. The number of colonies containing more than 50 cells was determined. Results were the average of three independent experiments.

### Construction of mouse xenograft model

HGC-27/FAF1 or HGC-27/NC cells (1 × 10^7^) in 0.2 ml of PBS were injected subcutaneously into the right armpit of male nude mice. Tumor diameter was measured every 3-4 d for 4 weeks. Tumor volume (mm^3^) was estimated by measuring the longest and shortest diameter of the tumor and applying the formula: volume = (shortest diameter)^2^ × (longest diameter) × 0.5.

### Histology

Immunohistochemistry was used to detect FAF1 protein expression. Samples of gastric cancer tissue, adjacent tissue and normal gastric tissue were fixed in 10% neutral-buffered formalin at room temperature for 48 h and embedded in paraffin. Tissue sections were incubated with goat polyclonal antibody against FAF1 (1:100; Santa Cruz, CA, USA), followed by peroxidase-conjugated rabbit anti-goat secondary antibody (1:500; Cell Signaling Technology). Signal was detected using colorimetry based on 3,3-diaminobenzidine (DAB). Xenograft sections from mice injected with HGC-27/FAF1 or HGC-27/NC cells were analyzed in the same way.

### ELISA of proinflammatory cytokines

Cells (1 × 10^5^ per well) were cultured in 6-well plates, and transfected with RNAi constructs targeting IKKβor p65 or with the negative-control RNAi construct. Transfected cells were then infected with *H. pylori* for 24 h at a multiplicity of infection of 100:1. Levels of TNF-α and IL-8 secreted by infected cells were determined using a commercial ELISA (KeyGen BioTech, Nanjing, China) according to the manufacturer's instructions. Absorbance at 450 nm was measured using an ELISA plate reader.

### Statistical analysis

All data were expressed as mean ± standard deviation (SD) and analyzed using SPSS 16.0 (IBM, Chicago, IL, USA). Inter-group differences in the rate of FAF1 positivity were assessed for significance using the chi-squared test; inter-group differences in survival curves, using the log-rank test; and inter-group differences in mRNA or protein levels, using one-way ANOVA or the Kruskal-Wallis H test, followed by the SNK post hoc test. Differences in tumor growth and volume were assessed for significance using multivariate analysis. Experiments were performed three times independently. Statistical significance was defined as P < 0.05.

## SUPPLEMENTARY TABLES







## Abbreviations

FAF1: Fas-associated factor 1
H. pylori: Helicobacter pylori
IL-8: interleukin-8
NF-κB: nuclear factor-κB
TNF-α: tumor necrosis factor-α
