# Supplementary material for: Fas-associated factor 1 inhibits tumor growth by suppressing Helicobacter pylori-induced activation of NF-κB signaling in human gastric carcinoma
Source: Oncotarget. 2016 Dec 20;8(5):7999–8009. doi: 10.18632/oncotarget.14033 (PMC5352377; doi:10.18632/oncotarget.14033)
Supplement: Supplementary file 1 [file oncotarget-08-7999-s001.pdf]

Fas-associated factor 1 inhibits tumor growth by suppressing Helicobacter *pylori*-induced activation of NF-κB signaling in human gastric carcinoma

SUPPLEMENTARY TABLES

Supplementary Table 1: Ingenuity pathway analysis (IPA) to analysis of canonical pathway, diseases/molecular function and network

See Supplementary File 1

Supplementary Table 2: Protein net summary report generated by Mascot for group FAF1 with H.pylori or without H.pylori

See Supplementary File 2
